# Influence of device-assisted suction against resistance (Mueller maneuver) on image quality in CTPA for suspected lung embolism

**DOI:** 10.1007/s00330-023-09834-3

**Published:** 2023-06-20

**Authors:** Niklas von Münchhausen, Sonja Janssen, Daniel Overhoff, Johann S. Rink, Bram Geurts, Andreas Gutzeit, Mathias Prokop, Stefan O. Schoenberg, Matthias F. Froelich

**Affiliations:** 1 Department of Radiology and Nuclear Medicine, University Medical Center Mannheim, Heidelberg University, Theodor-Kutzer-Ufer 1-3, 68167 Mannheim, Germany; 2 Department of Diagnostic and Interventional Radiology and Neuroradiology, Bundeswehr Central Hospital Koblenz, Koblenz, Germany; 3 Department of Department of Radiology and Nuclear Medicine, Radboud University Medical Center, Geert Grooteplein Zuid 10, 6525 GA Nijmegen, The Netherlands; 4 Department of Radiology, Paracelsus Medical University, Salzburg, Austria; 5 Department of Health Sciences and Medicine, Universität Luzern, Frohburgstrasse 3, 6002 Lucerne, Switzerland

**Keywords:** Pulmonary embolism, Pulmonary artery, Radiology

## Abstract

**Objectives:**

To investigate the effect of a device-assisted suction against resistance Mueller maneuver (MM) on transient interruption of contrast (TIC) in the aorta and pulmonary trunk (PT) on computed tomography pulmonary angiogram (CTPA).

**Methods:**

In this prospective single-center study, 150 patients with suspected pulmonary artery embolism were assigned randomly with two different breathing maneuvers (Mueller maneuver (MM) or standard end-inspiratory breath-hold command (SBC)) during routine CTPA. The MM was performed using a patented prototype (Contrast Booster™) which allows both the patient by means of visual feedback and the medical staff in the CT scanning room to monitor whether the patient is sucking sufficiently or not. Mean Hounsfield attenuation in descending aorta and PT was measured and compared.

**Results:**

Overall, patients with MM showed an attenuation of 338.24 HU in the pulmonary trunk, compared to 313.71 HU in SBC (*p* = 0.157). In the aorta, the values for MM were lower compared to SBC (134.42 HU vs. 177.83 HU, *p* = 0.001). The TP-aortic ratio was significantly higher in the MM group at 3.86 compared to the SBC group at 2.26, *p* = 0.001. TIC phenomenon was absent in the MM group, whereas it was present in 9 patients (12.3%) in the SBC group (*p* = 0.005). Overall contrast was better on all levels for MM (*p* < 0.001). The presence of breathing artifacts was higher in the MM group (48.1% vs. 30.1%, *p* = 0.038), without clinical consequence.

**Conclusions:**

Performing the MM with the application of the prototype is an effective way of preventing the TIC phenomenon during i.v. contrast-enhanced CTPA scanning compared to the standard end-inspiratory breathing command.

**Clinical relevance:**

Compared to standard end-inspiratory breathing command, the device-assisted Mueller maneuver (MM) improves contrast enhancement and prevents the transient interruption of contrast (TIC) phenomenon in CTPA. Therefore, it may offer optimized diagnostic workup and timely treatment for patients with pulmonary embolism.

**Key Points:**

*• Transient interruption of contrast (TIC) may impair image quality in CTPA.*

*• Mueller Maneuver using a device prototype could lower the rate of TIC.*

*• Device application in clinical routine may increase diagnostic accuracy.*

## Introduction

The clinical picture of pulmonary artery embolism secondary to deep vein thrombosis falls under the term venous thromboembolism (VTE) [1]. VTE is a common cardiovascular event with increasing incidence [2]. The fact that acute pulmonary artery embolisms (PE) may be asymptomatic or show only non-specific symptoms is a challenge. Symptoms of PE include sudden onset of dyspnea, chest pain, tachypnoea, hemoptysis, and the occurrence of dizziness or syncope [1].

In case of a suspected PE, laboratory testing of D-Dimer is often performed. In this context, the sensitivity of different point-of-care tests is reported as above 90%, however, specificity ranges from 42 to 64% [3]. As a result, D-Dimer can only be employed for the rule out of PE. Therefore, D-Dimer may be employed in the setting of intermediate and low risk for rule out. However, in case of a positive D-Dimer result, or in the setting of a high clinical suspicion of PE, a further diagnostic workup is needed: For these patients, a specific diagnosis and estimation of severity of PE is of high importance for urgent initiation of therapy. In this context, CT angiography of the pulmonary arteries (CTPA) is reported to offer a sensitivity of 83% and specificity of 96% [4]. Therefore, CTPA is recommended in multiple guidelines [5], including the current recommendations of the European Society of Cardiology (ESC) [6] as the gold standard for patients with suspected PE. However, the diagnostic accuracy of CTPA may be reduced due to transient interruption of contrast (TIC) [6], a flow-related phenomenon of non-enhanced blood from the inferior vena cava entering the pulmonary vascular system and reducing attenuation of contrast and, subsequently, diagnostic performance. Therefore, from a physiology perspective, the TIC phenomenon may be interpreted as a mixing phenomenon of non-contrasted inflow of blood via IVC into the right heart—a mechanism proposed by Gosselin et al in 2004 [7] and further elaborated by Wittram C et al in 2007 [8]. The TIC phenomenon has been reported to be present in more than 20% of patients [9] and, therefore, represents a relevant clinical challenge in CTPA imaging. An absolute Hounsfield density of below 200 has been described as associated with TIC criteria in more than 70% of cases [9]. First studies have indicated that specific breathing maneuvers like “suction against resistance” [10] and “suction/inspiration against resistance” [11] might be able to overcome this problem. The “suction/inspiration against resistance” maneuver is also referred to as the modified Mueller maneuver (MM). The MM is defined as forced inspiration against a closed glottis [10, 12] which results in a decrease in IVC and an increase in SVC velocity [12]. Furthermore, scanner hardware, in particular, the utilization of high-pitch scanners, and scanning parameters do have a significant impact on image quality. Despite the more widespread use of high-pitch scanners, where breath-hold times and artifacts due to the inability to hold one’s breath can be reduced dramatically, the problem of TIC is not fully solved by shorter breath-hold times.

Therefore, the aim of our study was to investigate if a device-assisted breathing maneuver “suction against resistance” results in better contrast in CTPA target arteries and if the occurrence of the TIC phenomenon can be reduced.

## Materials and methods

### Sample size estimation

Based on a one-sided Fisher’s exact test and prevalence of TIC phenomenon of 5% in the MM (Mueller maneuver) group compared to 20% in SBC (standard breathing command) group, an assumed *α* = 0.05 and a targeted power of 0.8, a sample size of at least 142 patients was calculated.

### Patient collective and study design

In our prospective, institutional-review-board-approved (2019-400 M-§23bMPG) single-center study, patients with suspected pulmonary artery embolism were enrolled and randomly assigned into two groups (MM and SBC) with different breathing maneuvers during routine CTPA. Patients were enrolled based on the following criteria: Age  ≥ 18 years and clinical indication for CTPA due to suspected PE. Exclusion criteria were the inability of informed consent, i.v. access at the lower extremity, insurmountable language barriers, severe hearing impairment, very severe breathing impairment, or lack of compliance. Patients were randomized to either device-assisted MM or SBC 1:1. For all patients, written informed consent was obtained and evaluation of imaging results was performed after anonymization. The data was also evaluated with regard to subjective and objective image quality measures.

The MM was performed using a specially designed and patented prototype breathing assistance device system (Contrast Booster™ System, Ulrich GmbH & Co. KG) which allows both the patient and the examiner to monitor the correct performance of the Mueller maneuver throughout the CT scan.

### Computed tomography imaging acquisition and reconstruction

CTPA was performed on a 128-slice single-source CT scanner system (SOMATOM Definition AS + , Siemens Healthcare GmbH). Scanning was performed in the cranio-caudal direction. A total of 60 ml iodine-based contrast agent (75% Imeron 350, Bracco, 25% NaCl 0.9%) was applied with a flow rate at 4 mL/s followed by a NaCl chaser bolus of 30 mL) with bolus tracking with the region of interest (ROI) within the pulmonary trunk. CTPA data was reconstructed with a slice thickness of 1.5 mm and an increment of 1.2 with a medium-sharp convolution kernel recommended by the vendor for CTPA (Bf37).

### Device and inspiration maneuver

#### Group 1: Mueller maneuver

The Mueller maneuver was performed using a dedicated prototype Contrast Booster system (Contrast Booster™ System, Ulrich GmbH & Co. KG) which allows both the patient via bio-feedback and the radiological staff outside the scanner room to monitor the correct performance of the Mueller maneuver throughout the scan and interact with the patient at any time. The system consists of a patient interface unit (PIU) and two charger and communication units which are wirelessly connected via Bluetooth. The PIU consists of a battery, a pressure sensor, and a visual display. A green light centrally on the bio-feedback light-emitting diode (LED) band indicates that he or she is sucking at the correct intensity level. An orange or red light, on the other hand, indicates suction to be either too strong or too weak. The investigator in the control room sees the same visual feedback on the visual display of the Charger and communication unit (CCU) and instructs via microphone, the patient to start sucking shortly after i.v. contrast injection starts to generate a continuous negative pressure. At this moment there is a massive contraction of the diaphragm on the inferior vena cava, resulting in interruption of the venous uncontrasted blood flow from the abdominal cavity in the direction of the right atrium. At the same time, there is a significant increase in the flow of the superior vena cava, with which the bolus of contrast medium reaches the right atrium and thus the pulmonary artery in a highly concentrated form, without being diluted by the non-contrasted blood from the blood of the abdominal cavity [10].

To control breathing command during suction against resistance, the flow volume curve is displayed to the investigator on a tablet PC, Samsung Tab A (Samsung Electronics Co., Ltd.) with an Android operating system Version 5.1.1 (Open Handset Alliance).

#### Group 2: Standard breathing command

The control group had to follow a standard automated breathing command telling the patient just shortly before the scan started “Please breathe in and hold your breath” and after completion of the scan “Continue breathing.”

**Fig. 1:**
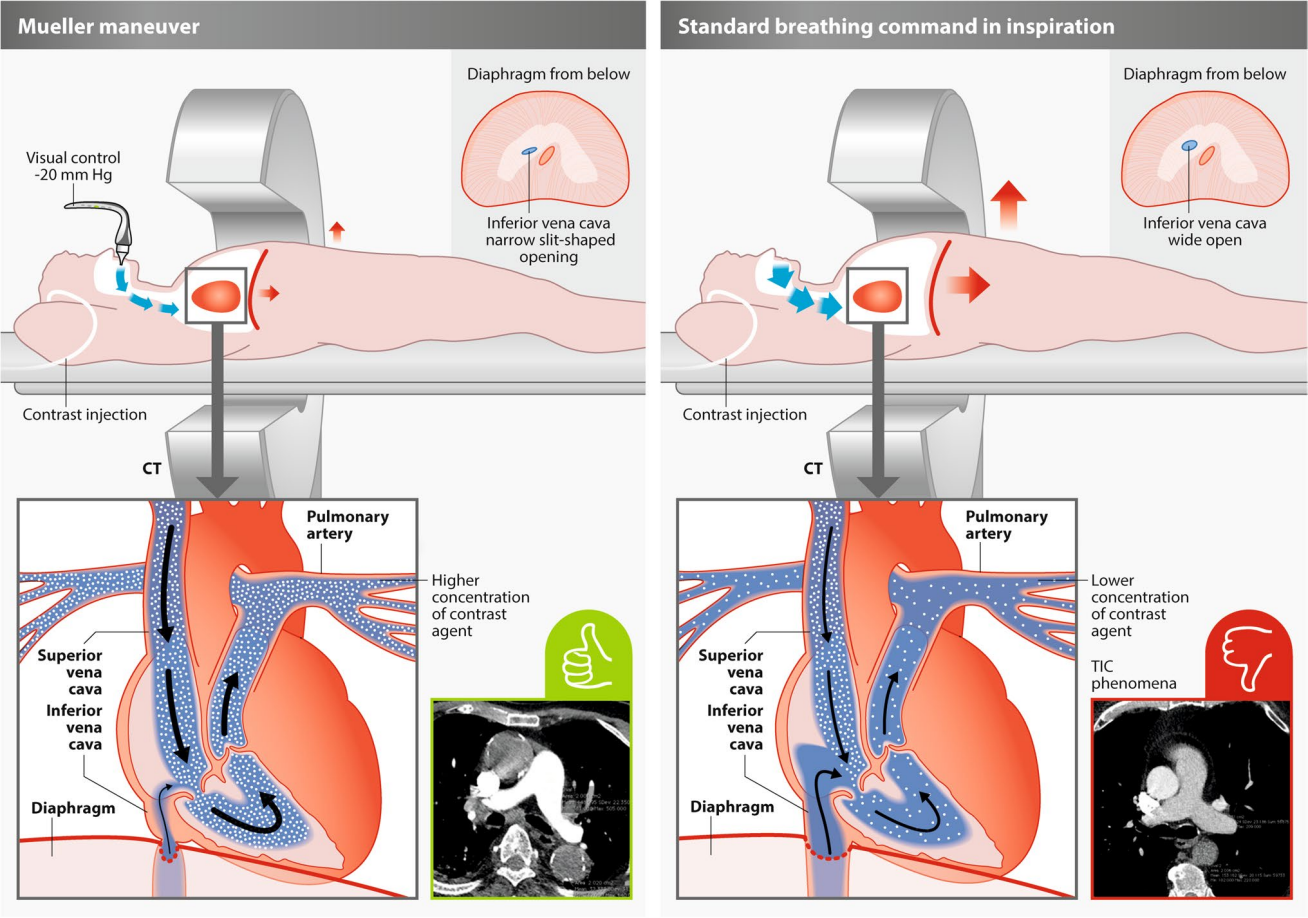
Mueller maneuver and standard breathing command: During the Mueller maneuver (left image), the patient sucks on the device, which allows a defined negative pressure (–20 mmHg). This causes the inferior vena cava to be compressed by the diaphragm due to breathing resistance. At the same time, there is a clear increase in the flow of the superior vena cava, which clearly concentrates the bolus of contrast medium, which is coming from the superior vena cava. Due to the lack of inflow of uncontrasted blood from the inferior vena cava, it reliably prevents a transient interruption (TIC) and concentrates the contrast bolus. During inspiration in the picture on the right, there is often a reverse flow behavior with an increase in the size of the inferior vena cava and an increased inflow of non-contrasted blood from the abdominal cavity. This often leads to the TIC phenomenon [9, 10].

The applied approach and complex physiological implications are summarized in Fig. 1.

### Image analysis

Image analysis was performed in a dedicated PACS-viewer solution (Aycan workstation PRO, Version 3.14.006, Aycan Medical Systems LLC). ROIs were placed in both the pulmonary trunk and the descending aorta at the transverse level of the pulmonary trunk by a radiology resident with 5 years of experience in CT imaging.

The study image quality analysis was performed analogous to the preliminary study on the incidence of the TIC phenomenon by Sudarski et al enrolled at the same institution [9]. In all CT data sets, the contrast attenuation was measured in HU (Hounsfield units) in the pulmonary trunk and in the descending aorta on the level of the pulmonary trunk. An ROI of 2 cm^2^ was generated in these two vessels. Measurements were only performed in artifact-free areas, without atherosclerotic plaques or thromboembolic material being present. Subsequently, the ratio of the measured density value in the pulmonary trunk and in the descending aorta was calculated. A ratio of  ≥ 1 was considered normal and a value  < 1 was considered a TIC phenomenon if contrast inflow via the SVC was detectable.

The presence of adequate contrast was evaluated at the level of the pulmonary trunk, lobar level, segmental level, and subsegmental level individually by the reader. Furthermore, the presence of relevant breathing artifacts and pulmonary embolism was evaluated. The presence of breathing artifacts was evaluated, and it was recorded whether these affected the diagnostic accuracy to a relevant degree. The evaluation of the artifacts was performed in a two-step approach: First, the evaluation was performed with respect to the contrast at all lung artery levels. This was followed by a second assessment of the presence of respiratory artifacts. Here, respiratory artifacts were defined as having a relevant detrimental effect on lung parenchyma evaluation. To exclude the influence of too strong respiratory artifacts on the contrast evaluation, a separate evaluation of patients with breathing artifacts was performed.

### Statistical analysis

Statistical analysis was performed with dedicated statistical analytics software (R statistics, version 4.1.0) [13]. R: A language and environment for statistical computing. R Foundation for Statistical Computing) with the packages readxl, tableone, and ggplot2. To compare means, t-test for unpaired samples was calculated. To compare categorical variables, chi-squared test was performed. A* p* value of below 0.05 was assumed to be statistically significant. The distribution of HU measurements was visualized in boxplots.

## Results

### Patient recruitment

**Fig. 2:**
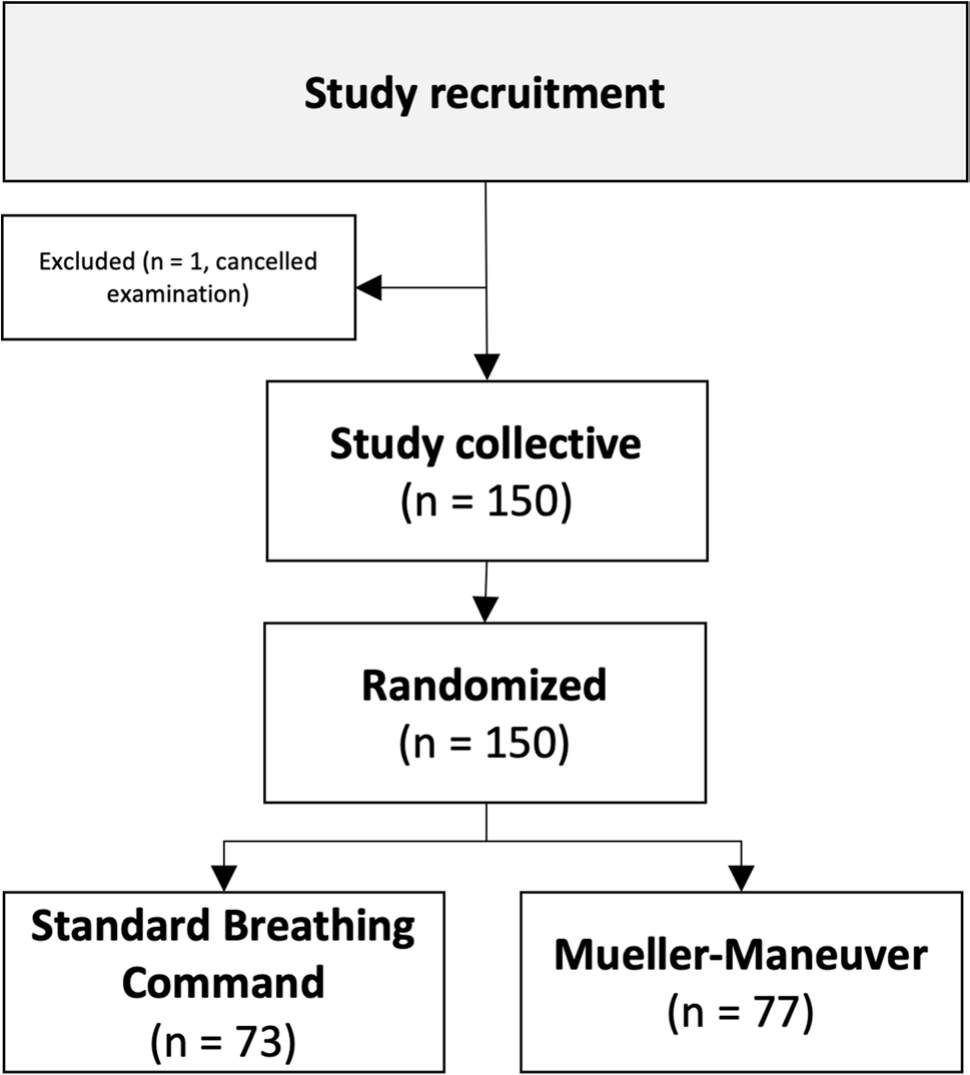
Prospective patient recruitment

**Fig. 3:**
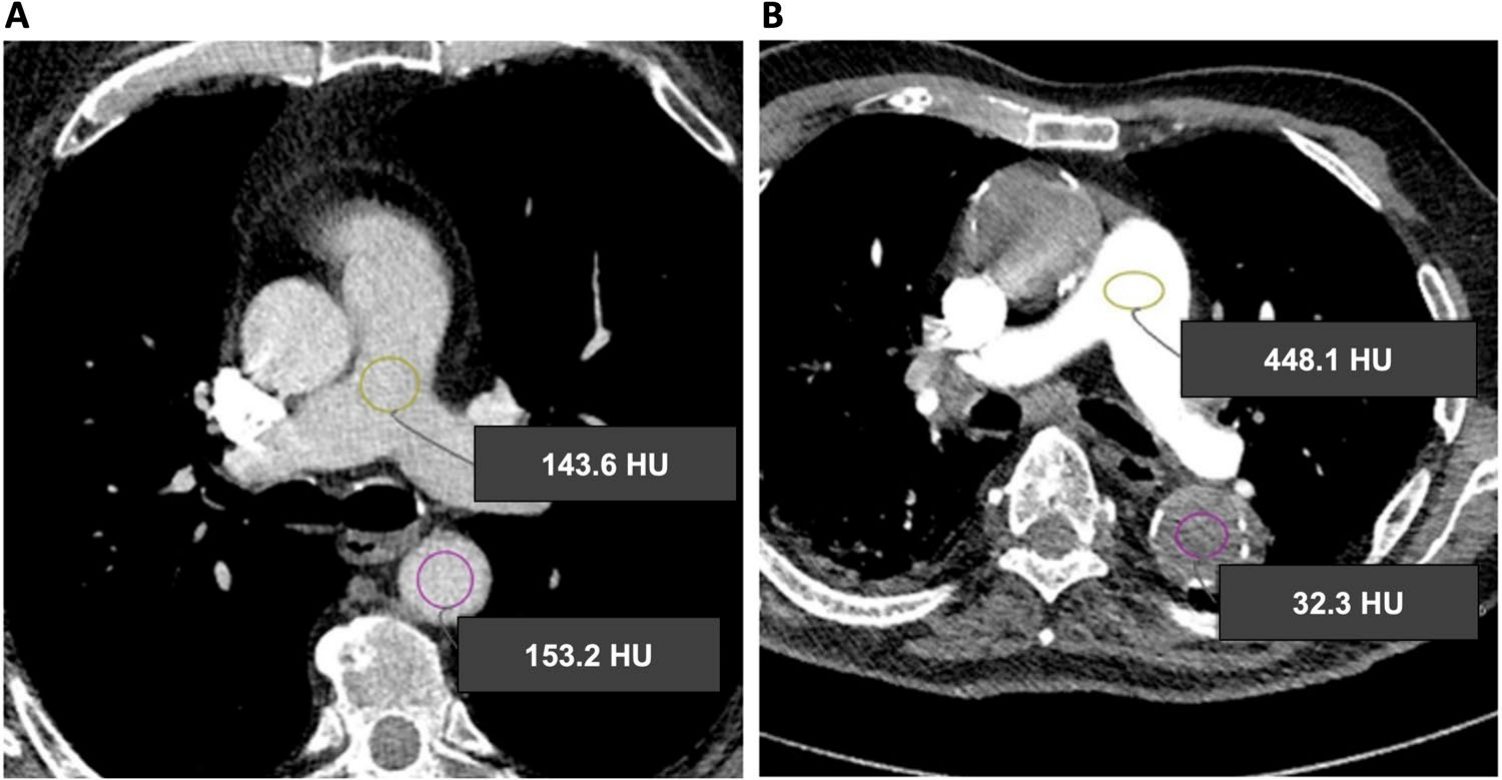
Example patients. **A  **A 75-year-old male patient with standard breathing command. PT/DA-ratio  < 1 with transient interruption of contrast. **B** A 86-year-old male patient with Mueller Maneuver, PT/DA-ratio  > 1

Based on the inclusion criteria, 151 patients each with a suspected pulmonary artery embolism were enrolled and randomly assigned to two study groups with different breathing maneuvers. One patient withdrew from the CTPA examination and the study at his own request before the CT scan was performed. Therefore, the final study collective consisted of 150 patients, which completed the study examination successfully, regardless of their group assignment. In the MM group, the device could be applied in all cases. A group comparison in terms of artifacts and image quality was performed based on the whole collective without further exclusion of patients. The study inclusion workflow is summarized in Fig. 2. An example patient is demonstrated in Fig. 3.

### Comparison of Mueller maneuver and standard breathing command

There were no significant differences in age, sex, and rate of pulmonary embolism between both study groups (all *p* > 0.05). In the MM group, the rate of fully diagnostic image quality, defined as optimal diagnostic contrast in combination with the absence of relevant other artifacts such as movement/breathing, was higher at 89.6% compared to 60.3% (*p* < 0.001). Correspondingly, contrast at all levels investigated separately was higher in the MM group (all *p* < 0.01). However, slight breathing artifacts without consequence were more common in the MM group with 48.1% compared to 30.1% in SBC (*p* = 0.038). Specifically, a separate evaluation of these cases with breathing artifacts reducing the diagnostic accuracy in the lung parenchyma (*n* = 59) did not show an influence of these artifacts on lung embolism evaluation. The group comparison results are summarized in Table 1. Pulmonary embolism was present in 5 cases (6.5%) in the MM group compared to 7 cases (9.6%) in the SBC group (*p* = 0.691).

**Table 1:** Patient collective and group comparison

	Level	Overall	Mueller-M	Standard	*p*
Number of patients		150	77	73	
Sex (%)	Female	69 (46.0)	35 (45.5)	34 (46.6)	1.000
	Male	81 (54.0)	42 (54.5)	39 (53.4)	
Age (mean (SD))		63.39 (16.92)	63.83 (14.95)	62.93 (18.88)	0.746
Treatment (%)	Mueller maneuver	77 (51.3)	77 (100.0)	0 (0.0)	< 0.001
	Standard procedure	73 (48.7)	0 (0.0)	73 (100.0)	
HU in pulmonary trunc (mean (SD))		326.30 (105.92)	338.24 (102.07)	313.71 (109.12)	0.157
HU in descending aorta (mean (SD))		155.55 (79.48)	134.42 (72.77)	177.83 (80.64)	0.001
HU ratio pulmonary trunc / descending aorta (mean (SD))		3.08 (2.94)	3.86 (3.56)	2.26 (1.77)	0.001
Presence of lung embolism (%)	No	138 (92.0)	72 (93.5)	66 (90.4)	0.691
	Yes	12 (8.0)	5 (6.5)	7 (9.6)	
Transient interruption of contrast	No	141 (94.0)	77 (100.0)	64 (87.7)	0.005
(%)	Yes	9 (6.0)	0 (0.0)	9 (12.3)	
Diagnostic contrast on all levels (%)	No	37 (25.0)	8 (10.4)	29 (39.7)	< 0.001
	Yes	111 (75.0)	69 (89.6)	44 (60.3)	
Central contrast in diagnostic quality (%)	No	11 (7.4)	1 (1.3)	10 (13.7)	0.009
	Yes	138 (92.6)	76 (98.7)	63 (86.3)	
Lobar contrast in diagnostic quality (%)	No	15 (10.1)	2 (2.6)	13 (17.8)	0.005
	Yes	134 (89.9)	75 (97.4)	60 (82.2)	
Segmental contrast in diagnostic quality (%)	No	29 (19.5)	4 (5.2)	25 (34.2)	< 0.001
	Yes	120 (80.5)	73 (94.8)	48 (65.8)	
Subsegmental contrast in diagnostic quality (%)	No	40 (26.8)	10 (13.0)	30 (41.1)	< 0.001
	Yes	109 (73.2)	67 (87.0)	43 (58.9)	
Breathing artifacts (%)	No	91 (60.7)	40 (51.9)	51 (69.9)	0.038
	Yes	59 (39.3)	37 (48.1)	22 (30.1)	

**Fig. 4:**
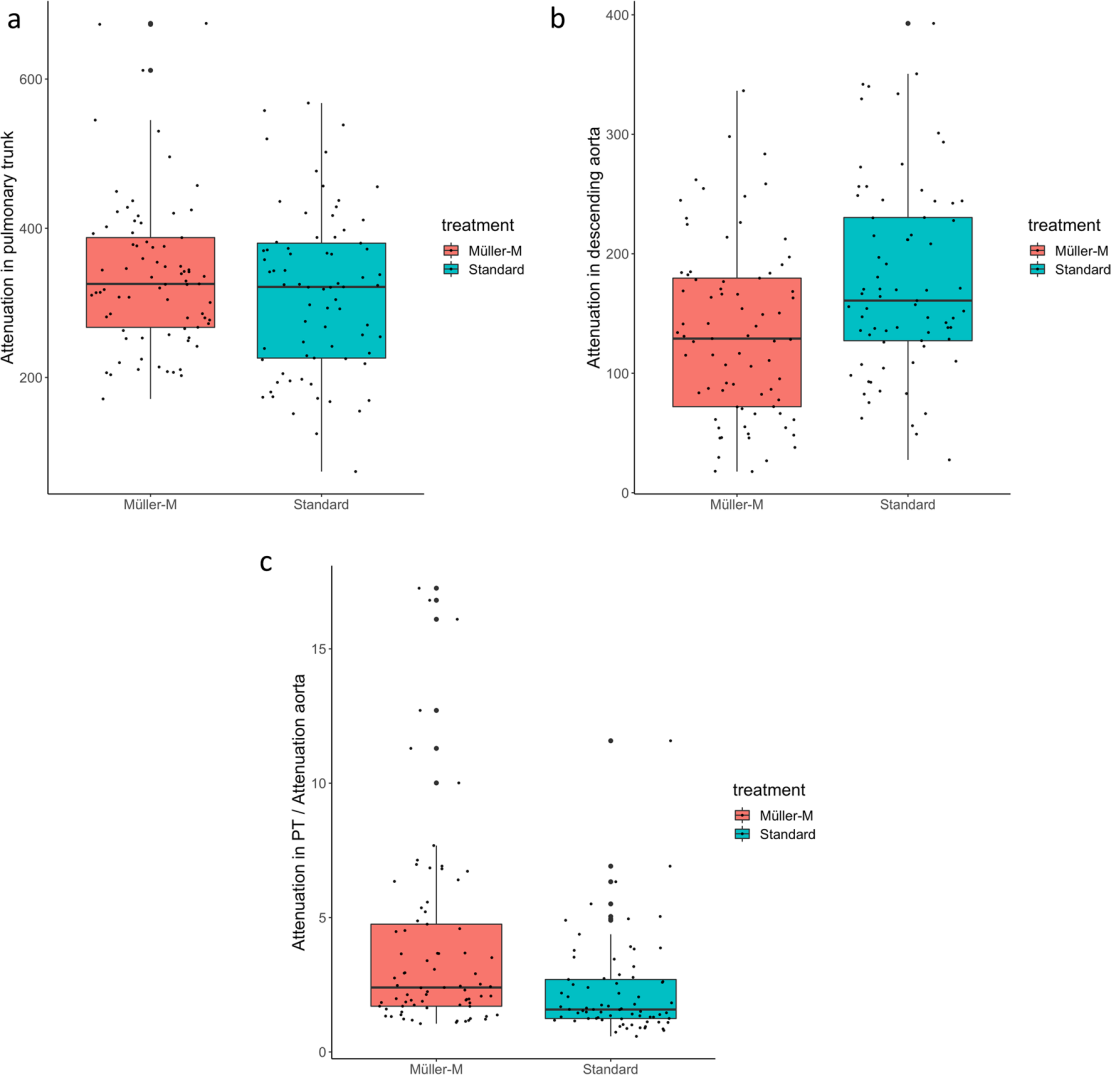
Comparison between device vs no device. **a** Attenuation in the pulmonary trunk. **b** Attenuation in descending aorta. **c** Attenuation in pulmonary trunk / attenuation in the aorta

The mean CT attenuation in the pulmonary trunk was 338.24 HU (SD 102.07) in the MM group compared to 313.71 HU (SD 109.12) in the SBC group (*p* = 0.157, Fig. 4a). The minimum contrast measured in the pulmonary trunk in the MM group was 171.21 HU compared to 73.88 HU in the SBC group. The contrast in the descending aorta was 134.42 (SD 72.77) HU for MM compared to 177.83 (SD 80.64) for SBC (*p* = 0.001, Fig. 4b). The TP-aortic ratio was 3.86 (SD 3.56) for MM compared to 2.26 (SD 1.77) for SBC (*p* = 0.001, Fig. 4c). TIC phenomenon was not present in the MM group (0 cases) compared to 9 cases (12.3%) in the SBC group (*p* = 0.005).

## Discussion

This prospective study showed that the performance of the device-assisted Mueller maneuver (MM) compared to the standard breathing command (SBC) did in tendency increase contrast attenuation in the pulmonary trunk in CTPA studies. Furthermore, the presence of the TIC phenomenon was reduced significantly to 0% in this single-center study, and using the device resulted in a better overall contrast on the central, lobar, segmental, and subsegmental levels. As a result, an increase in diagnostic accuracy in patients with suspected pulmonary embolism might be feasible by device-assisted Mueller maneuver.

Given that the rate of TIC phenomenon was significantly lower in the MM group, lower blood flow from the IVC [7] and higher blood flow into the pulmonary artery system due to negative pressure [10] might be regarded as potential explanations for the better diagnostic quality in the MM group. This may confirm the positive effect of this technique, which was first described by physiology in Fig. 1 and in 2014 [9, 10]. This results in clear contrast of the contrast medium bolus in the pulmonary artery and prevents the inflow of unconcentrated blood from the abdominal cavity (TIC). Furthermore, a slightly higher proportion of patients with breathing artifacts in this study may reduce diagnostic accuracy, especially in basal areas of the lungs. However, an association with reduced contrast of the pulmonary arteries was not found in this study.

The concern of suboptimal contrast has been discussed since the implementation of CTPA in clinical routine, as it poses a relevant clinical challenge. Therefore, the influence of different breathing techniques during CTPA scanning was investigated in several studies. Gosselin et al found the TIC phenomenon as an inspiration-associated artifact in CTPA in 37.2% of patients enrolled [7]. Therefore, expiration and breath-hold before CTPA was investigated. Mortimer et al compared expiratory and inspiratory CTPA examinations and found significantly higher attenuation in the pulmonary trunk (*p* < 0.05) for expiratory CTPA [14]. However, they found a lower parenchymal image quality for expiratory CTPA, due to difficulties for patients to hold their breath in end-expiration because the feeling of suffocation is more dominant compared to an end-inspiratory breath-hold. The superior contrast of expiration was confirmed in further research [15]. From this experience, the suction against resistance maneuver was proposed in order to achieve optimal CTPA contrast while maintaining lung parenchyma contrast. An MRI feasibility study performed by one of the co-authors of this research article showed a significant alteration of the blood flow ratio of SVC and IVC by application of a device-assisted suction against resistance approach [10]. These findings were further supported in another prospective pilot study of 15 patients for CTPA [11]. A monocentric study by Manava et al investigated device-assisted breathing maneuvers for CTPA with a different device but did not investigate the presence of the TIC phenomenon [16]. Their conclusions in favor of resting inspiratory position are in contradiction to earlier published evidence and to the physiologic considerations mentioned above. Manava et al published an erratum stating that the device applied was “custom-made” and was not performed with the patented conformité européenne (CE)–certified Contrast Booster™ System [17].

The results of our study presented in this paper are in line with the MR- and CTPA-pilot studies mentioned above and support the assumption that Mueller-Maneuver assisted by an appropriate device can increase contrast attenuation in CTPA and reduce the presence of TIC phenomenon. The clinical importance of this finding is supported by evidence showing that the TIC phenomenon is present in every fifth patient using standard end-inspiratory breath-hold command [9]. However, with high-end high-pitch scanners, it may be possible to perform CTPA without breathing commands at all while patients continue shallow breathing during the whole scan. This approach may, due to the physiological pressure settings within the chest, avoid TIC as well. Yet, it would be limited to such high-end high-pitch scanner systems.

The results presented in this work must be interpreted with regard to certain limitations: Blinding the readers for the presence or absence of the breathing device was not fully feasible, due to a variation in arm positioning and visibility in the topogram. Certain device-associated artifacts may be possible: In this study, breathing artifacts did differ significantly between MM and SBC groups for the collective. Yet, this may be addressed by techniques, which do not require the device to be held in the hand. Furthermore, the applicability of the device may differ based on the patient’s condition. However, in this study, the device was applied to a diverse collective of patients in a university medical center emergency setting. Additional studies on sub-collectives or in different clinical settings may be of interest.

In summary, this work shows the advantages of a device-assisted, suction against resistance breathing maneuver (Mueller maneuver) in CTPA studies with regard to diagnostic image quality and, especially, mitigation of the TIC phenomenon in a prospective study. Implementation of an appropriate device in PE diagnostics with CTPA may be a feasible strategy to improve diagnostic accuracy and lower the risk of missed PE.

## Abbreviations

CCU: Charger and communication unit
CE: Conformité européenne
CT: Computed tomography
CTPA: Computed tomography pulmonary angiogram
ESC: European Society of Cardiology
HU: Hounsfield unit
IVC: Inferior vena cava
LED: Light-emitting-diode
MM: Mueller maneuver
MRI: Magnetic resonance imaging
PE: Pulmonary embolism
PIU: Patient interface unit
PT: Pulmonary trunk
ROI: Region of interest
SBC: Standard end-inspiratory breath-hold command
SVC: Superior vena cava
TIC: Transient interruption of contrast
VTE: Venous thromboembolism

## Declarations

### Guarantor

The scientific guarantor of this publication is PD Dr. Matthias Frölich.

### Statistics and biometry

No complex statistical methods were necessary for this paper.

### Study subjects or cohorts overlap

None of the study subjects or cohorts have been previously reported.

### Methodology

• prospective

• randomised controlled trial / diagnostic or prognostic study

• performed at one institution
